# Molecular Docking and Structure–Activity Relationship Study of Polyphenols with Antibacterial and Antibiotic-Modulating Properties

**DOI:** 10.3390/microorganisms14020281

**Published:** 2026-01-25

**Authors:** Hayat Trabsa, Imane Krache, Naouel Boussoualim, Anfal Kara, Nadhir Saouli, Mohammad Raish, Byong-Hun Jeon, Hyun-Jo Ahn, Yacine Benguerba

**Affiliations:** 1 Department of Nature and Life Sciences, Faculty of Natural and Life Sciences and Earth and Universe Sciences, University of Biskra, Biskra 07000, Algeria; hayat.trab@yahoo.fr (H.T.); saoulinadhir7@gmail.com (N.S.); 2 Department of Biochemistry, Faculty of Natural and Life Sciences, University Ferhat Abbas of Setif 1, Setif 19000, Algeria; doussakr@yahoo.fr; 3 Department of Microbiology, Faculty of Natural and Life Sciences, University Ferhat Abbas of Setif 1, Setif 19000, Algeria; naouel_24@yahoo.fr (N.B.); karaanfel98@gmail.com (A.K.); 4 Department of Pharmaceutics, College of Pharmacy, King Saud University, P.O. Box 2457, Riyadh 11451, Saudi Arabia; mraish@ksu.edu.sa; 5 Department of Earth Resources and Environmental Engineering, Hanyang University, 222 Wangsimni-ro, Seongdong-gu, Seoul 04763, Republic of Korea; hjahn93@hanyang.ac.kr; 6 Laboratoire de Biopharmacie et Pharmacotechnie (LBPT), University of Ferhat Abbas Setif 1, Setif 19000, Algeria

**Keywords:** polyphenols, propyl gallate, antibacterial activity, structure–activity relationship, molecular docking, β-lactamase

## Abstract

The antibacterial activity of 18 phenolic compounds, including flavonoids and phenolic acids, against organisms of *Escherichia coli*, *Klebsiella pneumoniae*, and *Proteus vulgaris* that are resistant to several drugs was assessed in this study using the agar diffusion method. The strain’s strong resistance was confirmed by antibiotic susceptibility testing, which used fourteen drugs and only found inhibition zones for five of them. Out of the polyphenols, four compounds were effective against *P. vulgaris*, five against *K. pneumoniae*, and twelve against *E. coli* bacteria. The greatest inhibitory zone (18.75 ± 0.25 mm) against *E. coli* was shown by propyl gallate, an ester of gallic acid. Activity was significantly impacted by structural changes. Propyl substitution increased antibacterial activities across all strains, while methoxy substitution decreased them. The antibacterial effectiveness was reduced by the hydroxylation of flavonoids and the C3–C4 dihydroxylation of cinnamic acid. Propyl gallate primarily had antagonistic effects, while combination experiments demonstrated additive, synergistic, and antagonistic interactions. Propyl gallate (ΔG = −7.5 kcal/mol) exhibited substantial binding affinities with TEM-1 and NDM-1 β-lactamases via hydrogen and hydrophobic interactions, according to molecular docking. These results demonstrate propyl gallate as a viable antibacterial adjuvant option and validate the structure–activity relationship of phenolic compounds.

## 1. Introduction

The therapeutic properties of natural substances seem to be the most discussed topic by the general public, especially with the fast progression in chemistry and technology, which pushed the pharmaceutical and agro-alimentary industries to obtain biologically active compounds from renewable natural sources [1]. The use of medicinal herbs as a primary source drew the attention of researchers to study the natural molecules, such as polyphenols, which differ in their biological structures and their properties. These medicinal plants are used for developing novel therapeutic agents, such as antibiotics [2].

Dietary polyphenols are increasingly studied for their protective effects on human health. They constitute a diverse group of phenolic compounds whose subgroups vary in stability, bioavailability, and biological activity. Key classes such as phenolic acids, flavonoids, and tannins contribute to their major pharmacological functions, including antioxidant, cardioprotective, anticancer, anti-inflammatory, and antimicrobial effects [3].

In nature, bacteria have many mechanisms of resistance, and they are at least effective against toxic molecules with which they are confronted in their environment [4]. The resistance of bacteria to antibiotics constitutes a global problem. For example, bacteria use ESBLs (extended-spectrum beta-lactamases) to become resistant to antibiotics. ESBLs are a type of enzyme or chemical produced by some bacteria [5]. One does not cease reading news on bacteria that are increasingly resistant to current antibiotics because of the abusive use of the latter. The study of the genetic and biochemical mechanisms makes it possible to better understand the molecular bases of resistance to antibiotics and the factors responsible for their dissemination. This resistance can be either natural or acquired [6].

The interactions between the phytochemical compounds of these plants can contribute significantly to the capacity of the natural extracts, which can present effects that are synergetic, antagonistic, or additive [7]. With the development in molecular biology, the target scientific research involves the purification of the active pure molecules. Because the compound bioactives seldom function independently, the synergic interactions are of vital importance to explain the difficulty of isolating only one active ingredient and of explaining the apparently low effectiveness of amounts of active components in the extracts of the plants [8].

In this context, the present study aims to evaluate the in vitro antibacterial activity of selected polyphenols, including flavonoids and gallic acid derivatives, and to investigate their structure–activity relationships. Molecular docking was employed alongside biological assays to gain mechanistic insights into compound–target interactions and to better explain the observed antibacterial effects.

## 2. Materials and Methods

The clinical bacterial strains of Gram-negative (ESBLs) include *Escherichia coli*, *Klebsiella pneumoniae*, and *Proteus vulgaris*, obtained from the laboratory of bacteriology and of parasitology, hospital of Hakim Saadane Biskra, Algeria, and identified by Dr. Khelil K. (specialist microbiology pharmacist doctor, Hakim Saadan hospital, Biskra, Algeria). All other reagents were purchased from Sigma Chemicals (Hamburg, Germany), Fluka (Buchs, Switzerland), and Prolab (Algiers, Algeria). All phenolic molecules (6 phenolic acids, 1 precursor, and 12 flavonoids) used in this study are from Sigma Aldrich, including phenolic acids (gallic acid, methyl gallate, propyl gallate, caffeic acid, chlorogenic acid, sinapic acid), flavonoids (quercetin, rutin, Catechin, catechin, epicatechin, naringenin, naringin, morin, flavone), and phenolic acid precursors (cinnamic acid).

### 2.1. Antibacterial Activity of Polyphenols

The following bacterial strains were used in the bioassays: *Escherichia coli*, *Klebsiella pneumoniae*, and *Proteus vulgaris*. The microorganisms were cultured overnight at 37 °C in nutrient agar. Suspensions of mid-exponential phase cultures with an optical density of 0.5 McFarland were made in isotonic sodium chloride solution. Petri dishes of sterile Mueller–Hinton agar were seeded with the appropriate bacterial suspension. Sterile blotting paper discs (6 mm in diameter) were used, onto which each phenolic compound was added (10 µL per disc); each chemical standard was dissolved in DMSO to a final concentration of 50 µg/mL. Two other sterile blank discs, one impregnated with 10 μL of water and one of DMSO, were used as negative controls. After incubation for 24 h at 37 °C, all plates were observed for zones of growth inhibition, and the diameter of these zones was measured in millimetres. For data analysis, the susceptibility of a bacterium towards flavonoids was tested by measuring the bacteriostatic diameter, the clear zone of inhibition around the discs, and comparing it to a known sensitivity drug [9]. So, each antibiotic and biomolecule will be presented by an individual practical value (PVi) of its antibacterial activity.

### 2.2. Phenolic Compounds and Antibiotics Interaction Test

All active phenolic compounds and antibiotics were used to evaluate the interaction between biomolecules and antibiotics. The test was carried out following the same steps described previously, except that in this test, 10 μL of biomolecules was added to the antibiotic discs for each strain. All possible combinations of 2 active compounds (antibiotic/biomolecule) were realized. After incubation for 24 h at 37 °C, the clear zone of inhibition around the discs was measured in mm to obtain the practical values of combination (PVc); the latter were compared to theoretical values (TVs). The theoretical value of combination (TVc) was calculated for each combination using the following equation:

$$TVc\left(mm\right)=(PViofantibiotic+\mathrm{PVi}\;\mathrm{of}\;\mathrm{biomolecule})\;-\;6$$

A difference (Dfc) was calculated by subtracting the results of the theatrical value of the combination from the practical values of the combination using the following equation:

Dfc=PVc−TVc

Presenting the results in this manner allowed us to easily distinguish those combinations that present an additive, antagonistic, or synergistic interaction.

### 2.3. Molecular Docking Study

Molecular docking analyses were performed on the most active molecule (propylegallate) to complement and validate the in vitro antimicrobial results. The simulations were carried out using the AutoDockTools-1.5.6 program. The selected bacterial target proteins were retrieved from the Protein Data Bank (PDB). For antibacterial activity, the proteins selected represent key enzymatic systems involved in essential bacterial metabolic pathways [10,11].

- Cell wall synthesis. D-alanine/D-alanine ligase (2ZDQ), penicillin-binding protein 1a (3UDI), and TEM-1 β-lactamase (1NYM).
- DNA and nucleotide synthesis. DNA gyrase (1KZN), topoisomerase IV (3RAE), dihydropteroate synthase (2VEG), and dihydrofolate reductase (3SRW).
- Protein synthesis. Isoleucyl-tRNA synthetase (1JZQ) and tyrosyl-tRNA synthetase (1X8X).

The docking grids were centered on the native ligand coordinates to encompass the active sites, with dimensions of 40 × 40 × 40 Å. To ensure an effective simulation, the ligand was initially prepared by optimizing it using the Chem3D program to achieve a stable geometry with minimum energy. Next, the receptors (proteins) were prepared using Discovery Studio. This involved removing water molecules and heteroatoms and adding polar hydrogen atoms and Kollman charges. Afterwards, molecular docking simulations were conducted using the AutoDock Vina program. The ΔG values reported correspond to the affinity scores predicted by AutoDock Vina, derived from its empirical scoring function. These scores were used for comparative purposes to select the most plausible docking poses and analyse ligand–target interactions. Docking free energies (ΔG) were converted into inhibition constants using Ki = exp(ΔG/RT) (R is the gas constant (1.985 × 10^−3^ kcal mol^−1^ K^−1^) and T is temperature in kelvins = 298.15 K) [12].

### 2.4. Statistical Analysis

The results are expressed as mean ± SD. Where applicable, the data were subjected to one-way analysis of variance (ANOVA), where Tukey’s multiple comparisons test determined the differences between extracts. Dunnett’s multiple comparisons test was used for comparisons between extracts and standards using the GraphPad Prism 10 program. *p* ≤ 0.05 was regarded as significant.

## 3. Results

### 3.1. Antibiogram of Strains

The strains showed a strong resistance against a high number (9) of tested antibiotics (Table 1). This extended spectrum of antibacterial resistance explains the use of these strains in this study to find new proposals as antibiotics, whereas the most active antibiotic against all tested strains was cefoxitin.

### 3.2. Antibacterial Activity of Phenolic Acids

Phenolic acids are plant- and fungi-derived secondary metabolites involved in defense against environmental stresses and biological threats. They also participate in allelopathic interactions [13]. Owing to their diverse pharmacological properties, phenolic acids can scavenge free radicals, chelate metal ions, and modulate enzymatic activities [14].

The screening of antibacterial activity of phenolic acids, using the agar disc diffusion method, showed that the inhibition diameters varied from 8 to 18 mm (Table 2).

The structures of cinnamic and benzoic acid derivatives used in this study are presented in Table 3.

### 3.3. Antibacterial Activity of Flavonoids

The results of the antibacterial activity are shown in Table 4. The general structures of tested flavonoids are shown in Table 5 [18,19].

### 3.4. Antibiotic–Flavonoid Antibacterial Effects

The rise of antibiotic-resistant bacteria has reduced the effectiveness of conventional treatments, creating an urgent need for new therapeutic strategies. Combining antibiotics with natural compounds or plant extracts offers a promising approach, and some of these combinations are already used clinically [12]. Molecular interactions in such mixtures can occur within the same plant (endointeractions) or between different plants or drugs (exointeractions). These interactions may be additive, synergistic, or antagonistic, depending on whether the combined effect equals, exceeds, or diminishes the activity of the individual components [23].

This part aims to evaluate the interaction between all active polyphenols and antibiotics. Table 6, Table 7 and Table 8 present the results of the combinations of polyphenols and antibiotics obtained by the disc diffusion method against *E. coli*, *P. vulgaris*, and *K. pneumoniae*, respectively.

Against *E. coli* (Table 6), the combinations tested were classified as antagonists, except for two combinations, naringenin/ amoxicillin + clavulanic acid and epicatechin/colistin, with a synergic and additive effect, respectively. Propyl gallate was the most active polyphenol against all tested bacteria. However, the latter presents the most antagonistic effect against *E. coli* when coupled with all polyphenols.

On the other hand, there is more variability of molecular interactions against *P. vulgaris* (Table 7). Synergic and additive effects are clearly abundant, where the naringenin + cefotaxime combination presents the most synergetic effect. The same effect was carried out with a combination of cefoxitin and all the tested compounds.

No positive effects were observed with *K. pneumoniae* (Table 8); all tested combinations present an antagonist effect.

### 3.5. Docking Study

Docking screening against a panel of bacterial targets revealed differential binding affinities, highlighting β-lactamases and cell wall-associated enzymes as the most favorable targets for the studied ligand. As summarized in Table 9, the strongest predicted binding was observed for TEM-1 (1NYM) and NDM-1 β-lactamase (4EXS), with docking energies of −7.6 and −7.5 kcal/mol, corresponding to low predicted inhibition constants (Ki = 2.69 and 3.18 µM, respectively). Similarly, D-alanine/D-alanine ligase (2ZDQ) exhibited favorable binding (−7.2 kcal/mol, Ki = 5.28 µM), suggesting potential interference with peptidoglycan biosynthesis. Moderate affinities were predicted for isoleucyl-tRNA synthetase (−7.0 kcal/mol) and tyrosyl-tRNA synthetase (−6.6 kcal/mol), whereas enzymes involved in DNA replication and folate metabolism, including DNA gyrase, topoisomerase IV, and dihydrofolate reductase, showed weaker binding energies and higher Ki values (≥14 µM). Notably, penicillin-binding protein 1a and GES-2 β-lactamase displayed comparatively low affinities, indicating a degree of selectivity toward metallo- and serine-β-lactamases rather than PBPs. Overall, this target profiling suggests that the ligand preferentially stabilizes within β-lactamase active sites, particularly NDM-1 and TEM-1, consistent with the detailed interaction analyses and supporting a mechanism of action centered on enzymatic inhibition rather than broad multi-target antibacterial activity.

**Figure 1:**
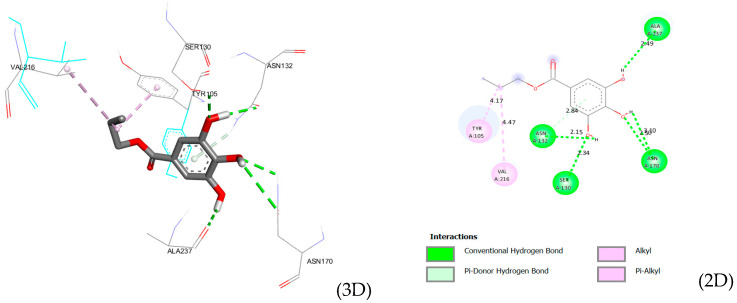
Interactions of propyl gallate with TEM-1 (1NYM) (2D and 3D). Green dashed lines indicate conventional hydrogen bonds, violet highlights represent alkyl and π–alkyl interactions, and colored circles denote interacting amino acid residues.

Docking analysis of the ligand within the active site of *TEM 1 β-lactamase* (PDB ID: 1NYM) reveals a binding mode stabilized by a dense hydrogen bond network complemented by hydrophobic contacts. As shown by the 2D and 3D interaction analyses (Figure 1), the ligand establishes several short conventional hydrogen bonds with key polar residues, notably SER130 (2.34 Å), ASN132 (2.15 Å), ASN170 (2.50 and 3.10 Å), and the backbone carbonyl of ALA237 (2.49 Å). Among these, the interaction with ASN132 exhibits the shortest distance, suggesting a strong anchoring role in maintaining ligand orientation. In addition, a π donor hydrogen bond between ASN132 and the aromatic ring at 2.84 Å further contributes to stabilizing the aromatic core within the binding pocket. Beyond polar interactions, hydrophobic contacts involving VAL216 (alkyl, 4.47 Å) and TYR105 (π alkyl, 4.17 Å) provide additional stabilization through nonpolar packing, particularly around the ester and aromatic regions of the ligand. Collectively, these interactions indicate that ligand binding to TEM 1 is governed by cooperative hydrogen bonding with polar residues surrounding the active site, supported by hydrophobic contacts that enhance pocket occupancy. This interaction pattern supports a stable docking pose and suggests that inhibition may arise primarily from strong anchoring within the active site region rather than direct covalent or catalytic residue engagement.

**Figure 2:**
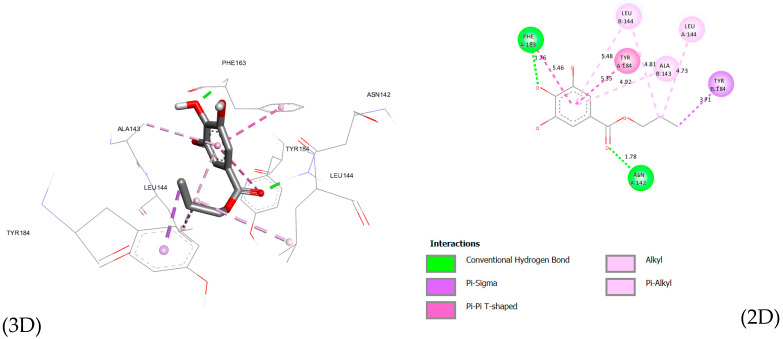
Interactions of propyl gallate with NDM-1 (4EXS) (2D and 3D). Green dashed lines indicate conventional hydrogen bonds, violet highlights represent alkyl and π–alkyl interactions, and colored circles denote interacting amino acid residues.

Docking of the ligand within the NDM-1 active site (PDB ID: 4EXS) indicates a stable binding mode supported by complementary polar and hydrophobic interactions (Figure 2). The ligand forms two short conventional hydrogen bonds with ASN142 and PHE163, with distances of 1.78 Å and 1.76 Å, respectively, providing strong anchoring within the pocket. The aromatic core is further stabilized by π-σ interaction with TYR184 (3.71 Å) and π-π T-shaped interactions with PHE163 and TYR184 (5.46 and 5.35 Å). In addition, the alkyl substituent extends toward a hydrophobic region defined by LEU144 and ALA143, generating multiple alkyl and π-alkyl contacts (4.73–5.48 Å) that enhance pocket occupancy. Overall, binding to NDM-1 is driven by a conserved hydrogen bonding framework complemented by aromatic stacking and hydrophobic packing, suggesting that substituent effects primarily modulate stabilization through nonpolar interactions rather than altering the core anchoring network.

## 4. Discussion

The in vitro activity of cefoxitin has been previously evaluated against *Mycobacterium abscessus* [12]. It is a β-lactam antibiotic derived from cephamycin C; its resistance to destruction by lactamases results in a broad spectrum of antibacterial activity, which includes anaerobic as well as Gram-positive and Gram-negative aerobic bacteria. The presence of the 7α-methoxy group in the nucleus confers upon cefoxitin a high degree of resistance to hydrolysis by β-lactamases produced by specific strains [23].

The methyl gallate did not produce inhibition zones against any of the tested bacteria, whereas caffeic and chlorogenic acids produced an inhibition zone only against *E. coli*. Many studies have shown the capacity of some bacterial strains (lactic acid bacteria) to metabolize phenolic acids by decarboxylation and/or reduction [15,24]. The results allow us to propose this activity to our resistant strains.

Propyl gallate, a gallic acid derivative, is highly active against various microbial strains. It is widely used as a synthetic antioxidant in foods, cosmetics, and packaging [16]. Phenolic acids are classified into hydroxybenzoic and hydroxycinnamic acids, based on C1–C6 and C3–C6 skeletons, respectively. Chemically, these compounds have at least one aromatic ring in which at least one hydrogen is substituted by a hydroxyl group [17].

The objective of this part of the study was to evaluate the antibacterial effect of phenolic acids on selected strains and to establish a structure–activity relationship. A simple comparison between the antibacterial activity values [25,26] and the chemical structures of acids was carried out. Gallic acid exhibits a moderate activity against two strains despite its high hydroxylation. Methyl gallate is a derivative of the gallic acids; however, it does not have any antibacterial activity [27].

Substitution of the C_7_ hydroxyl group (–OH) with the methoxy group (–OCH_3_) very significantly decreased (*p* < 0.0001) the activity of 3-4-5-trihydroxybenzoic acid against *E. coli* and *K. pneumoniae*, but not against *P. vulgaris*. Sa’nchez-Maldonado and his collaborators have demonstrated the same proposition against lactic acid bacteria [28].

However, the substitution of hydrogen and methyl groups with a propyl group (CH_2_CH_2_CH_3_) of C_7_ very significantly increased (*p* < 0.0001) the antibacterial activity of 3-4-5-trihydroxybenzoic acid against all strains.

Cinnamic acids are a group of aromatic carboxylic acids (C6–C3) appearing naturally in the plant kingdom. Many studies prove that most of the cinnamic acids, their esters, amides, aldehydes, and alcohols, show significant growth inhibition against one or several bacterial and fungal species. But, their structure-activity relationship was not established [29,30].

In the present study, cinnamic acid presents a moderate activity against all strains. However, gallic acid (a 3,4-dihydroxycinnamic acid) did not show any antibacterial activity against *K. pneumoniae* and *P. vulgaris*, but it has an activity comparable to cinnamic acid activity against *E. coli*. These results showed that C3-C4 dihydroxylation of cinnamic acid very significantly (*p* < 0.0001) decreases its antibacterial activity against *K. pneumoniae* and *P. vulgaris*. The same hydroxylation can increase, not significantly (*p* > 0.05), its antibacterial activity against *E. coli*. On the other hand, quinoylation of 3,4-dihydroxycinnamic acid significantly decreases (*p* < 0.006) the activity against *E. coli*. Sinapic acid activity does not present a significant difference (*p* > 0.05) compared to cinnamic acid against *K. pneumoniae* and *P. vulgaris*. But, a significant difference has been shown against *E. coli*.

The effect of hydroxyl (–OH) and methoxy (–OCH3) groups on the antimicrobial activity of phenolic acids was studied by a comparison of the inhibition zone of caffeic acid and sinapic acid. The results show that C3-C5 methylation of C3-C4 hydroxybenzoic acid can significantly increase the antibacterial effect against *P. vulgaris* and *K. pneumoniae* (*p* < 0.0004 and *p* < 0.0001, respectively). Conversely, it decreases the antibacterial effect against *E. coli* (very significantly, *p* < 0.0001).

Flavonoids, the most abundant dietary polyphenols with a C6–C3–C6 backbone, are vital in plant physiology and exhibit multiple biological activities, including antioxidant, anti-inflammatory, anticancer, and antimicrobial effects [31]. In vitro studies (Table 4) show variable antibacterial activity against multi-drug-resistant Gram-negative bacteria; naringenin is the only flavonoid active on all tested strains. The literature data indicate that standard bacterial strains are generally more sensitive to antibiotics and natural plant compounds than contemporary clinical isolates [32].

Flavonoids possess a fifteen-carbon skeleton comprising two benzene rings (A and B) connected by a heterocyclic pyran ring (C). They are classified into groups, such as flavones, flavonols, and flavanones, which differ in C-ring oxidation and substitution patterns, while individual compounds within each class vary in A- and B-ring substitutions [33,34].

Flavones have been widely investigated for their antibacterial activity, but in this study, the only active flavone is flavone. However, C3-hydroxylation of the C ring and C5,6-dihydroxylation of the A ring significantly enhanced the antibacterial activity of flavone against *E. coli* and *K. pneumoniae* (*p* < 0.0001 and *p* < 0.0002, respectively). From the above investigations, it follows that the increase in hydroxylation of flavone has no meaningful influence on its antibacterial activity. Many studies showed that flavone derivatives showed higher antibacterial effects than flavones such as chrysin against Gram-positive bacteria [34].

Furthermore, we observed that a number of hydroxyl groups at aromatic rings do not correspond with higher antimicrobial activity of flavonols, i.e., quercetin, fesetin, and morin have five, four, and five hydroxyl groups, respectively. But, they were not active against all tested bacteria. On the other hand, glycosylation of the hydroxyl groups at position C3 of quercetin results in improved antibacterial activity against *E. coli* (*p* < 0.0001).

Adamczak and their collaborators showed that the level of sensitivity of the bacterial species to plant substances is very diverse and strongly depends not only on the type of active compound but also on the selected strains; it may also affect significant discrepancies in the results between individual investigations [35].

Tested flavan-3-ol and flavanone are the most active flavonoids against *E. coli*. In the past, there has been confusion regarding nomenclature; flavanols are also referred to as flavan-3-ols or catechins. The predominant flavanols are catechin (3,5,7,3′,4′-pentahydroxyflavan) and epicatechin (3,5,7,3′,4′-pentahydroxyflavan) [31]. They are stereo-isomers of one another, differing in the configurational disposition related to the two and three positions of the benzopyran moiety [36]. This difference presents a significant (*p* < 0.0001) influence on the antibacterial activity against *E. coli,* where the catechin is the most active tested flavan-3-ol. It is well recognized that tea catechins have preventive effects against cancer, heart disease, diabetes, and age-related diseases. Some bacteria could catalyze C-ring cleavage of flavan-3-ol, such as catechin and epicatechin [37,38]. This is possibly the reason why catechin and epicatechin did not exhibit any activity against the tested strains, *P. vulgaris* and *K. pneumoniae.*

The effect of glycosylation (Rutinose) on the antimicrobial activity of hydroxyflavanone was studied by a comparison of the inhibition zone of naringenin and naringin. The results show that C7-glycosylation of 5-7-4′ Trihydroxyflavonone can significantly increase the antibacterial effect against *P. vulgaris* and *K. pneumoniae* (*p* < 0.0001).

A simple comparison between catechin and quercetin showed that the double bond between C2 and C3 and/or the C4 carbonyl group of the C heterocycle can inhibit the antibacterial activity. Another comparison between naringenin and chrysin structures showed that the double bond between C2 and/or C3 of heterocycle C and C4′-hydroxylation of the B ring can significantly inhibit (*p* < 0.0001) the antibacterial effect against all tested bacteria. The inactivity of some flavonoids, which have a C4′-hydroxyl group of the B ring, such as quercitin, fisetin, and morin, puts a spot around the double bond between C2=C3 of heterocycle C, which can play a crucial role in the antibacterial activity against all tested bacteria.

Numerous studies have shown that polyphenols can synergize with antibiotics in vitro by inhibiting β-lactamase activity or bacterial topoisomerases, leading to cell death through DNA synthesis inhibition, growth arrest, and multiple double-stranded DNA breaks [39].

Many studies reinforce the point that free radicals accelerate the lethal activity of antimicrobials. Sublethal concentrations of antimicrobials can be mutagenic, probably via free radical accumulation, because antibiotic-induced free radicals lead to the production of 8-oxo-guanine, which is mutagenic. Thus, antioxidants may contribute in two ways to the emergence of antimicrobial resistance: reduction in lethal activity and increased recovery of mutants [40]. This hypothesis can explain the total antagonism between the used polyphenols (potent antioxidant agents) and antibiotics.

The molecular docking analysis provided mechanistic insights into the antibacterial activity of propyl gallate, which emerged as the most potent compound against the tested bacterial strains. Among the tested enzymes, the *TEM-1 β-lactamase* mutant (PDB: 1NYM) and *NDM-1 β-lactamase* (PDB: 4EXS) exhibited the strongest affinities. These results are consistent with recent studies highlighting the antimicrobial potential of phenolic compounds. Liu et al. (2025) demonstrated that propyl gallate can restore tigecycline activity by inhibiting Tet (X4) enzymes in vitro, in vivo, and in silico [41]. Similarly, Pestana-Nobles et al. (2022) showed that polyphenolic compounds displayed strong binding to β-lactamases, surpassing some classical inhibitors in docking scores [11].

This study combines experimental antibacterial evaluation with structure-based in silico analysis to provide mechanistic insight into the activity of gallate derivatives. The results demonstrate that propyl gallate displays enhanced biological performance driven primarily by increased hydrophobic character and improved pocket occupancy while preserving a conserved hydrogen bonding framework within key β-lactamases, such as *NDM-1* and *TEM-1*. Docking analyses indicate that substituent effects are mainly associated with hydrophobic stabilization rather than the formation of new polar interactions, underscoring the importance of physicochemical contributions alongside enzyme-level binding. Overall, these findings strengthen the structure–activity understanding of phenolic esters and support their relevance as promising scaffolds for the development of resistance-modulating or adjunct antibacterial agents.

## 5. Conclusions

This study demonstrated that polyphenols, especially phenolic acids, such as propyl gallate, possess significant antibacterial activity against multi-drug-resistant Gram-negative bacteria, whereas flavonoids displayed comparatively weaker effects. The structure–activity relationship analysis highlighted that a single structural rule does not determine antibacterial efficacy but rather depends on multiple factors, including bacterial strains, including bacterial strain specificity and the chemical subgroup of polyphenols. Notably, the presence of a C7-methoxy group reduced the activity of hydroxybenzoic acids against *E. coli* and *K. pneumoniae*. In contrast, C3–C4 dihydroxylation in cinnamic acids diminished their activity against *K. pneumoniae* and *P. vulgaris*. In flavonoids, glycosylation exhibited variable effects, enhancing activity in the case of C7-glycosylated hydroxyflavanones but reducing it with C3-glycosylated hydroxyflavonols. Furthermore, the double bond between C2=C3 in the heterocyclic ring was identified as a key determinant of antibacterial potential.

The enhanced in vitro antibacterial activity of propyl gallate is likely driven by its increased hydrophobicity, which favors membrane permeation and intracellular accumulation, in addition to its polyphenolic redox activity. While the docking results indicate favorable interactions with key enzymes, such as NDM-1 and TEM-1, these in silico findings alone cannot fully account for the observed biological effects. Instead, the experimental activity appears to arise from a combination of physicochemical membrane-related effects and enzyme-level interactions, highlighting a multi-factorial mechanism of action.

However, the evaluation of polyphenol–antibiotic combinations revealed variable outcomes, ranging from synergistic to antagonistic interactions, with naringenin combined with cefotaxime showing the strongest synergy against *P. vulgaris*. Taken together, these findings highlight the promise of polyphenols, particularly phenolic acids, as potential antibacterial agents and adjuvants to conventional antibiotics. Nonetheless, the variability of their interactions with standard drugs underscores the need for further in vivo validation and pharmacological assessments before clinical translation.

## Figures and Tables

**Table 1:** Inhibition zone (mm) of antibiotics presented by individual practical values (PVi) against the tested strains.

Antibiotics	*E. coli*	*P. vulgaris*	*K. pneumoniae*
Amoxicillin + clavulanic acid	14.50 ± 0.70	17.50 ± 0.70	24.00 ± 0.70
Cefotaxime	10.00 ± 0.84	15.50 ± 0.70	12.00 ± 0.28
Ampicillin	/	/	/
Tetracycline	/	/	/
Streptomycin	/	12.50 ± 0.70	/
Clindamycin	/	/	/
Ceftazidime	/	/	/
Colistin	12.75 ± 0.35	/	16.25 ± 0.35
Fusidic acid	0	/	/
Gentamicin	24.00 ± 0.70	25.00 ± 0.70	19.75 ± 0.35
Penicillin	/	/	/
Oxacillin	/	/	/
Cefoxitin	26.25 ± 0.35	25.50 ± 0.70	30.50 ± 0.75
Vancomycin	/	/	/

All PVIs were expressed as mean ± SD of triplicates.

**Table 2:** Inhibition zone (mm) of phenolic acids presented by individual practical value (PVi) against the tested strains.

Phenolic Acids	*E. coli*	*P. vulgaris*	*K. pneumoniae*
Gallic acid	8 ± 0.28	/	8.25 ± 0.35
Methyl gallate	/	/	/
Propyl gallate	18.75 ± 0.35	11.75 ± 0.35	10 ± 0.42
Caffeic acid	10 ± 0.70	/	/
Chlorogenic acid	8.75 ± 0.35	/	/
Sinapic acid	8.25 ± 0.35	7.25 ± 0.35	8.75 ± 0.35
Cinnamic acid *	9.75 ± 0.35	7.75 ± 0.35	8.75 ± 0.35

* is a precursor of phenolic acids. All PVIs were expressed as mean ± SD of triplicates.

**Table 3:** Chemical structures of phenolic acids used in this study: [15,16,17,18,19,20].

Precursors		R_1_	R_2_	R_3_	R_4_	R_5_
Benzoic acid 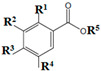	Gallic acid		OH	OH	OH	
	Methyl gallate		OH	OH	OH	CH_3_
	Propyl gallate		OH	OH	OH	CH_2_CH_2_CH_3_
Cinnamic acid 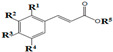	Caffeic acid			OH	OH	
	Chlorogenic acid			OH	OH	5-Quinoyl
	Sinapic acid		OCH_3_	OH	OCH_3_	

All R gaps represent a hydrogen atom.

**Table 4:** Inhibition zone (mm) of flavonoids presented by individual practical value (PVi) against the tested strains.

Flavonoid	*E. coli*	*P. vulgaris*	*K. pneumoniae*
Flavone	9.50 ± 0.70	/	7.25 ± 0.35
3-Hydroxyflavone	/	/	/
Chrysin	/	/	/
Diosmin	/	/	/
Quercetin	/	/	/
Rutin	9.25 ± 0.35	/	/
Fisetin	/	/	/
Morin	/	/	/
Catechin	10.00 ± 0.35	/	/
Epicatechin	8.25 ± 0.35	/	/
Naringenin	9.75 ± 0.35	7.75 ± 0.35	7.75 ± 0.35
Naringin	9.00 ± 0.70	/	/

All values were expressed as mean ± SD of triplicates.

**Table 5:** Chemical structures of the flavonoids used [20,21,22].

Subgroups	Molecule	Substitution							Others	
		3	5	6	7	2′	3′	4′	C_2_=C_3_	C_4_=O
Flavone 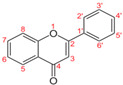	Flavone								+	+
	3-Hydroxyflavone	OH							+	+
	Chrysin		OH		OH				+	+
	Diosmin		OH		O-Rut		OH	OCH3	+	+
Flavonol 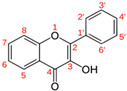	Quercetin	OH	OH		OH		OH	OH	+	+
	Rutin	O-Rut	OH		OH		OH	OH	+	+
	Fisetin	OH			OH		OH	OH	+	+
	Morin	OH	OH		OH	OH		OH	+	+
Flavan-3-ol 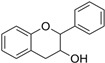	Catechin	OH	OH		OH		OH	OH	−	−
	Epicatechin	OH	OH		OH		OH	OH	−	−
Flavanone 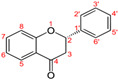	Naringenin		OH		OH			OH	−	+
	Naringin		OH		O-Rut			OH	−	+

C2=C3: double bond between C2 and C3 of heterocycle C, C4=O: C4 carbonyl group of the C heterocycle, (+): presence, (−): absence, Rut: Rutinose.

**Table 6:** Inhibition zone (PVc: mm) of combinations between polyphenols + antibiotics against *E. coli* and differences (Dfc: mm) between theoretical and practical values.

ATB Molecule	Amoxicillin + Clavulanic Acid		Cefotaxime		Colistin		Gentamicin		Cefoxitin	
	PVc	Dfc	PVc	Dfc	PVc	Dfc	PVc	Dfc	PVc	Dfc
Gallic acid	10.75	−5.75	11.00	−1	14.00	−0.75	21.50	−4.5	26.00	−2.25
Propyl gallate	10.75	−16.5	12.25	−10.5	14.50	−11	22.50	−14.25	11.25	−27.75
Caffeic acid	11.50	−7	8.00	−6	14.25	−2.5	21.00	−7	23.25	−7
Chlorogenic acid	10.50	−6.75	10.25	−2.5	14.75	−0.75	21.75	−5	22.75	−6.25
Sinapic acid	10.00	−6.75	9.50	−2.75	14.75	−0.25	21.75	−4.5	19.25	−9.25
Cinnamic acid *	11.50	−6.75	9.75	−4	14.75	−1.75	21.00	−6.75	19.00	−11
Flavone	13.00	−5	9.00	−4.5	13.50	−2.75	21.00	−6.5	26.75	−3
Rutin	12.50	−5.25	10.50	−2.75	8.25	−7.75	21.25	−6	21.75	−7.75
Catechin	11.00	−7.5	10.25	−3.75	15.00	−1.75	22.00	−6	26.20	−4.05
Epicatechin	9.75	−7	8.50	−3.75	15.00	0	21.50	−4.75	25.50	−3.4
Naringenin	20.75	+2.5	9.75	−4	14.75	−1.75	21.75	−6	20.50	−9.5
Naringin	11.75	−5.75	11	−2	14.50	−1.25	22.50	−4.5	25.50	−3.75

* is a precursor of phenolic acids. All values were expressed as the mean of triplicates, and all SD ≤ 0.25. (+) synergic effect, (−) antagonistic effect, and (0) additive effect.

**Table 7:** Inhibition zone (PVc: mm) of combinations between polyphenols + antibiotics against *P.vulgaris* and differences (Dfc: mm) between theoretical and practical values.

ATB Molecule	Amoxicillin + Clavulanic Acid		Cefotaxime		Streptomycin		Gentamicin		Cefoxitin	
	PVc	Dfc	PVc	Dfc	PVc	Dfc	PVc	Dfc	PVc	Dfc
Propyl gallate	14.25	0	35.25	0	13.50	−4.75	35.25	+4.5	29.50	−1.75
Sinapic acid	18.75	0	36.75	0	14.75	+1	35.25	+9	31.50	+4.75
Cinnamic acid *	12.75	0	38.25	0	11.50	−2.75	36.50	+9.75	31.50	+4.25
Naringenin	18.25	−1	36.25	+19	9.75	−4.5	34.75	+8	29.75	+2.5

* is a precursor of phenolic acids. All values were expressed as the mean of triplicates, and all SD ≤ 0.25. (+) synergic effect, (−) antagonistic effect, and (0) additive effect.

**Table 8:** Inhibition zone (PVc: mm) of combinations between polyphenols + antibiotics against *K. pneumoniae* and the differences (Dfc: mm) between theoretical and practical values.

ATB Molecule	Amoxicillin + Clavulanic Acid		Cefotaxime		Colistin		Gentamicin		Cefoxitin	
	PVc	Dfc	PVc	Dfc	PVc	Dfc	PVc	Dfc	PVc	Dfc
Gallic acid	22.75	−3.5	13.75	−0.5	15.12	−3.75	19.25	−2.75	28.75	−4
Propyl gallate	14.25	−13.75	12.50	−3.5	16.75	−3.5	21.75	−2	30.00	−4.5
Sinapic acid	23.75	−3	8.75	−6	17.75	−1.25	19.50	−3	26.75	−6.5
Cinnamic acid *	23.50	−3.25	10.75	−4	15.50	−3.5	20.50	−2	29.25	−4
Naringenin	24.50	−1.25	10.50	−3.25	14.50	−3.5	20.5	−1	30.25	−2

* is a precursor of phenolic acids. All values were expressed as the mean of triplicates, and all SD ≤ 0.25.

**Table 9:** Binding free energy values were computed by molecular docking propyl gallate to bacterial enzymes.

Target	Code pbd	Energy (kcal/mol)	Ki (µM)
IV topoisomerase	3RAE	−5.7	66.35
DNA gyrase	1KZN	−6	39.99
Dihydropteroate synthase	2VEG	−5.4	110.10
Dihydrofolate reductase	3SRW	−6.3	24.10
D-alanine/D-alanine ligase	2ZDQ	−7.2	5.28
Penicillin-binding protein 1a	3UDI	−5.3	130.34
GES-2 beta-lactamase	4QU3	−6.1	33.78
NDM-1 beta-lactamase	4EXS	−7.5	3.18
M182T mutant of TEM-1	1NYM	−7.6	2.69
Isoleucyl-tRNA synthetase	1JZQ	−7	7.40
Tyrosyl-tRNA synthetase	1X8X	−6.6	14.53

## Data Availability

All data generated or analyzed during this study are included in this published article.
